# Myocardial Viability: What We Knew and What Is New

**DOI:** 10.1155/2012/607486

**Published:** 2012-09-05

**Authors:** Adel Shabana, Ayman El-Menyar

**Affiliations:** ^1^The Department of Clinical Medicine, Weill Cornell Medical College, P.O. Box 24144, Doha, Qatar; ^2^The Department of Cardiology, Hamad Medical Corporation, P.O. Box 3050, Doha, Qatar; ^3^Clinical Research, Trauma Surgery Unit, Hamad General Hospital, P.O. Box 3050, Doha, Qatar

## Abstract

Some patients with chronic ischemic left ventricular dysfunction have shown significant improvements of contractility with favorable long-term prognosis after revascularization. Several imaging techniques are available for the assessment of viable myocardium, based on the detection of preserved perfusion, preserved glucose metabolism, intact cell membrane and mitochondria, and presence of contractile reserve. Nuclear cardiology techniques, dobutamine echocardiography and positron emission tomography are used to assess myocardial viability. In recent years, new advances have improved methods of detecting myocardial viability. This paper summarizes the pathophysiology, methods, and impact of detection of myocardial viability, concentrating on recent advances in such methods. We reviewed the literature using search engines MIDLINE, SCOUPS, and EMBASE from 1988 to February 2012. We used key words: myocardial viability, hibernation, stunning, and ischemic cardiomyopathy. Recent studies showed that the presence of viable myocardium was associated with a greater likelihood of survival in patients with coronary artery disease and LV dysfunction, but the assessment of myocardial viability did not identify patients with survival benefit from revascularization, as compared with medical therapy alone. This topic is still debatable and needs more evidence.

## 1. Introduction

Coronary artery disease (CAD) remains a principal cause of morbidity and mortality worldwide [1]. Many subjects with heart failure and underlying CAD have an important amount of viable but dysfunctional myocardium, where akinetic or severely hypokinetic myocardium keeps the ability to contract if perfusion improves [2]. This reawakening of myocardium after restoration of blood flow was noted as early as 1978 by Diamond et al. who referred to such myocardium as “hibernating,” a term popularized later by Rahimtoola and by Braunwald and Rutherford who emphasized the need for its identification and therapy through revascularization [3–5]. We reviewed the literature using search engines MIDLINE, SCOUPS, and EMBASE from 1988 to February 2012 using key words: myocardial viability, hibernation, and stunning, and ischemic cardiomyopathy. We found 230 pertinent articles including 45 non-English, 100 reviews, and 130 studies. The current paper summarizes the pathophysiology, methods, and impact of detection of myocardial viability, concentrating on recent advances in such methods.

## 2. Pathophysiology

The first view of adaptation involves dedifferentiation or embryonic regression, the so called “smart heart” hypothesis [6]. The process of adaptation is linked with a down-regulation in energy utilization, evidenced by a decrease in the expression of mitochondrial oxidative enzymes, and an upregulation of stress proteins [7]. This counterbalances the effects of ischemia but at the cost of an attenuated level of contractile function [8, 9]. The alternative is that this is not adaptation, but “forced degeneration.” Supporting this is the finding that hibernating myocardium also contains apoptotic cells and cells with autophagosomes, lysosomes, and vacuoles [10]. Whatever the cause, structural remodeling would be essential to restore contractility, thus chronically impaired but viable myocardium may take weeks or months to recover once flow is restored [11]. Interventions that bring back blood flow to the hibernating myocardium may return the myocytes to their physiologic function and reprogram the cells to normal expression of key proteins [12].

Stunning is another form of reversible segmental myocardial dysfunction in the setting of normal myocardial perfusion. Heyndrickx et al. [13] observed from canine experiments that “the myocardium rendered ischemic, but not irreversibly damaged, exhibits prolonged depression of regional myocardial function, long after the complete return of blood flow and resumption of a normal electrocardiographic pattern". One of the vital differences between these two experimental concepts is that resting myocardial perfusion is normal/near normal in stunning, but is reduced in hibernation [6, 14].

Classically, hibernation was thought to occur with sustained hypoperfusion, especially during tachycardia but with adequate residual flow to allow survival of the tissue in the lack of contractile activity [15]. In contrast to the extremely low flow states required to induce hibernation in animal studies, human studies suggest that hibernating tissue may have 70 to 80% of normal coronary flow [16]. However, the normal or near-normal blood flow at rest in hibernating segments is associated with impaired coronary flow reserves. As a result, these segments may be subject to recurrent episodes of ischemia (caused by increased demand when the tissue has a loss of coronary flow reserve), which eventually lead to a state of persistent postischemic dysfunction [17]. In other words, hibernation is the summation of repetitive and cumulative stunning, resulting in an apparent chronic reduction in left ventricular (LV) function [18].

## 3. Morphology

It was originally assumed that the recovery of function when hibernating myocardium is revascularized must indicate that structural changes are absent or minimal, as had been found in experimental models of stunned myocardium. However, since the early 1980s, it has been known that chronically dysfunctional myocardial segments demonstrate distinct morphological changes that can be verified by both the light and the electron microscope [6].

One prominent feature of the changes seen in cardiomyocytes, by light microscope, is the loss of contractile material, which is replaced with an amorphous, strongly Periodic Acid-Schiff (PAS)-positive material typical of glycogen in addition to variable intracellular fibrosis [19, 20]. There is a combination of normal, atrophied, and hypertrophied myocytes, with or without evidence of necrosis. Electron microscope shows loss and (or) disorganization of myofilaments and changes in the sarcoplasmic reticulum and mitochondria. These structural changes may contribute to slow functional recovery on revascularization [21]. Extracellularly, there may be increases in the quantity of collagen fibrils, elastic fibers, and fibroblasts [22]. However, significant fibrosis may not be present [7].

## 4. Evaluation of Viable Myocardium

The differentiation of viable from nonviable myocardium is therefore highly relevant in patients who are being considered for revascularization [23]. Many patients who demonstrate viability associated with severe LV dysfunction may still be candidates for revascularization rather than for cardiac transplantation [24].

### 4.1. Electrocardiography (ECG)

Q waves on the ECG were originally thought to indicate full-thickness myocardial infarction (MI), but in fact, there is no relationship between the presence and extent of Q waves after MI and infarct size assessed by myocardial perfusion imaging, and up to 60% of regions with Q waves have viable myocardium detected by imaging techniques [25]. Fragmented QRS complex has been suggested as a marker of scar but was not validated in other studies [26, 27]. ST-segment elevation at rest in leads with Q waves is associated with more severe wall-motion abnormalities, less contractile reserve and greater end-systolic volume. In the extreme case, this is seen as the persistent ST elevation of aneurysm formation [28]. In contrast, ST elevation developing during exercise is a marker of maintained viability [29]. Exercise induced ST segment elevation in infarct-related leads was found to have 82% sensitivity and 100% specificity in detection of viability by FDG (fluorodeoxyglucose) uptake [30]. Moreover, the presence of reciprocal ST-segment depression in addition to exercise-induced ST segment elevation indicate residual tissue viability with 84% sensitivity and 100% specificity patients with previous MI and 1-vessel disease [31]. To predict improvement of LV function after revascularization, exercise induced ST segment elevation with pseudonormalization of negative T waves in infarct-related leads had sensitivity of 80% and specificity of 89% [32]. After MI, a low QT dispersion of ≤70 msec had sensitivity of 83% and specificity of 71% to predict residual viability [33].

### 4.2. Imaging Techniques

Ventriculography is the oldest imaging technique and is rarely used clinically today [34, 35]. Other techniques depend on different characteristics of dysfunctional but viable myocardium. The most established and clinically used techniques include the following [36, 37]:

- nuclear imaging by Positron Emission Tomography (PET) (evaluating labeled FDG uptake),
- nuclear imaging by Single-photon emission-computed tomography (SPECT) (evaluating perfusion, cell membrane integrity, and intact mitochondria with thallium or technetium-labeled agents),
- echocardiography with dobutamine (to assess contractile reserve),
- echocardiography with intravenous contrast agents (to assess perfusion),
- in addition, MRI with dobutamine (to assess contractile reserve), and MRI or CT with intravenous contrast agents (to assess scar tissue) are emerging as promising techniques.

It is obvious that each of these techniques can detect viability by assessing different factors of the myocardial tissue.

## 5. Recent Trends in Viability Assessment

### 5.1. Positron Emission Tomography

 The strength of PET as an imaging technique relies on the versatility of positron emitting radionuclides that can be integrated into important biochemical molecules. Not only can the distribution of these molecules be imaged, but their uptake can be quantified. In this way, it is possible to assess myocardial perfusion, glucose utilization, fatty acid uptake and oxidation, oxygen consumption, contractile function, and presynaptic and postsynaptic neuronal activity [38].

Angiotensin converting enzyme (ACE) inhibitors have been radiolabeled and used in experimental systems to study the tissue ACE receptor system directly. Preliminary observations in explanted hearts from patients who had heart failure showed a relationship between binding of [18F]fluorobenzoyl-linsinopril and collagen replacement; ACE was absent in the collagen-stained areas and was increased in the juxtaposed areas of replacement fibrosis [39]. The increased ACE in the juxtaposed areas of replacement fibrosis may be a stimulus for collagen replacement and remodeling. In case, the increase of ACE is reversible with ACE inhibitors, noninvasive imaging with PET would allow monitoring of changes in ACE patterns in vivo, which may reflect progression of disease and the effect of therapies before collagen replacement ensues [40].

### 5.2. Single-Photon Emission-Computed Tomography

SPECT imaging identifies viable and infarcted myocardium based on regional differences in radiotracer uptake, with segments classified as viable as a consequence of preserved mitochondrial function (technetium SPECT) or preserved membrane integrity (thallium SPECT) [41].

Technetium-labelled tracers have advantages over thallium, such as a shorter half-life with lower radiation exposure to the patient, a higher energy gamma emission that reduces soft-tissue attenuation, more flexibility in imaging times after stress, and the potential for ECG-gated acquisition. However, unlike thallium, technetium tracers have significant redistribution, which necessitates 2 inject ions of the tracer (exercise and rest) for typical stress-rest protocols either on the same day or in two different days [40]. This may carry disadvantages since uptake depends on both perfusion and viability, and viability may be underestimated in areas with reduced perfusion at rest. In contrast, thallium uptake is independent of perfusion once redistribution is complete [38]. Some studies have found the technetium agent, Tc-99 m-2-methoxyisobutylisonitrile (MIBI), to be inferior to thallium for identifying viability 42 but others have found the two to be comparable [42].

To enhance the ability of technetium SPECT imaging to detect viability, several methods have been used; some are technical (e.g., quantitation of uptake and using ECG gating) and other included pharmaceutical additives (e.g., Nitrates and Trimetazidine).

Recently, Spadafora et al. [43] proposed a polar map of myocardial viability through gated SPECT. On baseline SPECT, the researchers obtained a parametric image of viable myocardium (VIA map) was obtained using a semi-automated method to subtract the point-to-point motion polar map from the perfusion polar map. The baseline motion polar map was subtracted from the motion polar ma p after revascularization to produce a parametric image of segments with functional recovery (REC). The VIA map was directly compared to the REC map to assess the ability of the VIA map to predict functional recovery after revascularization, it was directly compared to the REC map.

The VIA and REC maps were also represented as 3-D images. On the VIA map, segments with counts <20% of the peak activity were represented in black or dark blue indicating nonviable segments, whereas segments with counts ≥20% were considered hibernated. Similarly, on the REC map, segments with counts <20% of peak activity were represented in black or dark blue indicating regions without functional recovery, and segments with counts ≥20% of the peak were considered as showing functional recovery. The proposed polar map of myocardial viability obtained by gated SPECT imaging accurately predicts functional recovery after coronary revascularization [43].

Bisi et al. [44] proposed that nitrates might have a role in improving the ability of sestamibi imaging to predict myocardial viability. In some other studies, the addition of Trimetazidine to myocardial Tc-99 m sestamibi or tetrofosmin SPECT improved myocardial perfusion and LV function [45–47].

#### 5.2.1. SPECT with Fatty Acids

A variety of iodinated fatty acid compounds have been used to examine regional fatty acid metabolism in vivo [48]. Although many different fatty acids have been used for that purpose, most experience has been obtained with *β*-methyl-iodo-pentadecanoic acid (BMIPP) labeled with iodine-123, since it is metabolically trapped in the myocardium due to its methyl branching [49]. When myocardium is jeopardized by recurrent stunning, resting perfusion, or hibernation, glucose is metabolized in preference and a defect on fatty-acid imaging appears. The presence of metabolic embarrassment could be assumed If the defect is more intense than expected from the amount of viable myocardium assessed by a viability tracer such as thallium [38]. The viability BMIPP mismatch pattern has been shown to correspond with thallium redistribution and preserved contractile reserve after MI [50].

#### 5.2.2. Imaging of Innervation

A number of labeled analogues of noradrenaline have been investigated, but the most commonly used is iodine-123 metaiodobenzylguanidine (MIBG) [51]. In case of heart failure secondary to ischemic heart disease or cardiomyopathy, decreased MIBG uptake is a poor prognostic sign indicating advanced disease with denervation [52].

### 5.3. Hybrid and Gamma Camera Approaches

PET imaging is not widely available because of its expense and complexity. Even when a PET camera is available, imaging may be restricted to FDG because the half lives of ^13^N and ^15^O are too short to allow imaging without an on-site cyclotron. Thus, FDG imaging for myocardial viability has been combined with SPECT tracers. This hybrid approach has proved successful [53]. It is now also possible to image FDG using a conventional gamma camera, either using high-energy SPECT protocol or gamma camera PET protocol [54, 55].

### 5.4. Estimation of the Effective Radiation Exposure

The Effective radiation dose to patients varies broadly among different nuclear imaging techniques. The effective dose ranges from almost 2 mSv for standard PET scan modalities using ^13^N ammonia and ^15^O water studies to about 10 mSv for standard rest-stress protocols using 99 mTc sestamibi or tetrofosmin, rising over 20 mSv for ^201^thallium imaging protocols and approaching 30 mSv in dual isotope studies. Furthermore, the effective radiation dose of a 64-slice CT coronary angiography scan is nearly equal to that of a 99 mTc Myocardial Perfusion imaging study but lower than ^201^thallium scan [56].

### 5.5. Echocardiography

Echocardiography can allow detection of myocardial viability with a rather reasonable accuracy, using different techniques, that is, resting echocardiography, contrast echocardiography, tissue characterization and myocardial velocity imaging, and pharmacological stress echocardiography [14].

Dysfunctional, but viable myocardium can still preserve a contractile reserve, which may be evoked by an appropriate stimulus [14]. In patients with jeopardized but viable myocardium, the LV ejection fraction (EF) will show improvement with low-dose dobutamine in direct proportion to the number of segments with contractile reserve [56]. Dobutamine-induced segmental and global functional recovery correlates well with other, more complex imaging techniques, including PET and thallium scintigraphy [57, 58]. Furthermore, new developments in stress echocardiography can help as adjuvant to improve viability detection. These include contrast echocardiography, tissue Doppler imaging (TDI) and strain, and three-dimensional echocardiography.

#### 5.5.1. Myocardial Contrast Echocardiography (MCE)

Although dobutamine stress echocardiography (DSE) has excellent specificity for the identification of hibernating myocardium, its sensitivity tends to be lower than the other imaging modalities. The addition of MCE to DSE has been investigated in the effort to improve the diagnostic accuracy of echocardiography for prediction of viability in patients with chronic ischemic heart disease [59]. In addition to improving endocardial border detection during echocardiography, MCE has an important role in evaluation of myocardial perfusion [37, 60]. The concept underlying this is that myocardial perfusion is essential for cellular viability, thus detection of preserved myocardial microvasculature could differentiate between viable and dead myocardium. MCE uses intravenously injected microbubbles that cross the pulmonary vascular bed, to reach the myocardial segments. The presence of microbubbles in a myocardial segment suggests viability while non-enhancement of the contrast estimates the absence of significant viability in that segment [59]. Microbubble velocity and myocardial blood flow, assessed by MCE, were found to be the most significant quantitative parameters for prediction of contractile reserve after MI [61].

Data showed that MCE is more accurate than DSE alone, thallium SPECT, nitrate-enhanced technetium SPECT and PET in detection of viability [61–64]. Tousek et al. [65] reported that MCE had similar sensitivity but higher specificity to delayed-enhanced MRI.

#### 5.5.2. TDI in Viability Assessment

The use of TDI for viability prediction at rest has been limited by its lack of site specificity because the segment of interest can be “tethered” by neighboring segments. Some TDI parameters including peak systolic velocity, isovolumetric contraction, and time-to-peak systolic velocity have not been shown to consistently predict functional recovery [66, 67]. TDI measurement of preejection velocity, however, has been shown to be predictive of viability [59].

Myocardial velocity analysis by TDI at rest and during dobutamine stimulation could allow assessment of myocardial viability [68–70]. Pulsed Doppler tissue velocity analysis has been performed on apical views with analysis of systolic tissue velocities confined to the basal segments. This approach allows assessment of viability for a whole ventricular wall from apex to base [71]. A recent study demonstrated that diastolic tissue velocities determined at rest are enough to differentiate viable from nonviable myocardium although they are affected by age [72].

Chan et al. [73] showed that Strain rate imaging can be used to differentiate subendocardial infarcts, which have a greater likelihood of benefit from revascularization, from transmural infarcts. A rise in peak systolic strain rate by more than 0.23/s from rest to dobutamine stress, using FDG PET, could predict viability with a sensitivity of 83% and specificity of 84% [66, 67, 74]. Analysis of diastolic function using myocardial-deformation imaging can be used to assess myocardial viability. Dyssynergic but viable myocardial segments demonstrated an increase in early diastolic E-wave and late diastolic A-wave strain rate whereas nonviable segments were less responsive to dobutamine stimulation [75]. Furthermore, few studies have shown that myocardial-deformation imaging performed only at rest is enough to determine myocardial viability [76].

Because TDI-based strain is largely influenced by the angle between the ultrasound beam and the myocardial wall, Speckle tracking (2-D strain) was used as a new technique that tracks frame-to-frame movement of natural acoustic markers, or speckles [77]. Local 2D tissue velocity vectors are derived from the spatial and temporal data of each speckle. Thus, a more accurate assessment of regional myocardial deformation and reliable analysis of the transmural extent of necrosis may be feasible. Automated function image algorithm is a novel method based on two-dimensional strain imaging that enables the simultaneous quantification of myocardial strain in different left ventricular segments, and also provides global longitudinal peak systolic strain (GLPS). GLPS during dobutamine stress was found to be a promising, objective index to assess myocardial viability on the early stage of MI [78].

Other new echocardiographic techniques are gaining interest in the last decade as an adjuvant to assess myocardial viability; these include 3D echocardiography during dobutamine stress [79] and Integrated Backscatter analysis [79, 80]. The latter method is independent of wall motion and is shown to predict contractile reserve in ischemic myocardial damage [80].

### 5.6. Magnetic Resonance Imaging (MRI)

MRI has distinctive unique ability to assess viable and infarcted myocardium by different techniques as a one-stop shop [81]. MRI techniques have the advantage of no ionizing radiation. Owing to its superior spatial resolution, CMR (cardiac magnetic resonance) has a unique capability to assess small infarcts and to measure the transmural extent of MI. Therefore, it can detect microinfarcts associated with successful coronary angioplasty, as well as the detection of subendocardial infarcts which could be missed by SPECT or do not exhibit a wall motion abnormality [82].

There are 3 main techniques to assess myocardial viability; resting MRI (to measure end diastolic wall thickness), dobutamine MRI (to evaluate contractile reserve), and contrast enhanced (delayed enhanced) MRI [DE-MRI] (to detect the extent and transmurality of scar tissue) [37, 83].

Assessment of resting wall thickness and thickening by resting cine-MRI can be used to assess viability. The underlying hypothesis is that regions of myocardial thinning reflect chronic myocardial infarction. The combination of wall thickness and systolic wall thickening tend to improve the sensitivity and specificity of the technique [84]. Cine-MRI performed during dobutamine infusion can be used to assess potential for contractile response to coronary revascularization with diagnostic performance at least comparable to dobutamine echocardiography and superior to it in those with poor acoustic windows [84].

**Figure 1:**
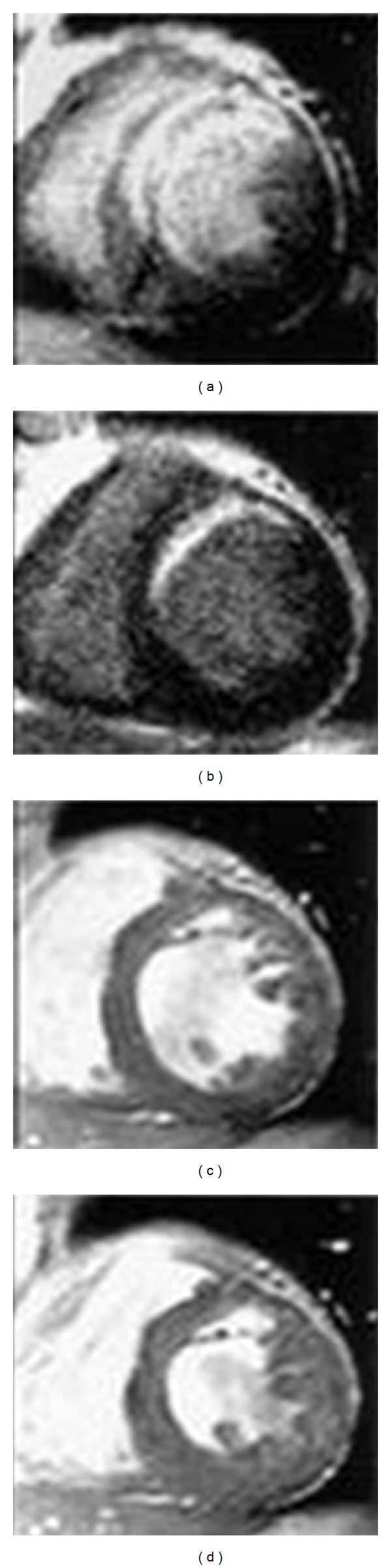
Contrast-enhanced magnetic resonance images in the acute state (a) and chronic state (b), and cine images in the chronic state (c, diastole; d, systole) in Patient who sustained an anteroseptal myocardial infarction. The contractility of the anteroseptal wall was not improved in the chronic state in this patient (with permission from Ichikawa et al.(2005), Am Coll Cardiol, Elsevier Inc., [90]).

DE-MRI can concomitantly detect infarcted and normal myocardium within a given myocardial segment and thereby allows extent of viability to be assessed [84]. The concept for this approach is that infarcted tissue accumulates gadolinium and appear as hyperenhanced or “bright” regions on T1-weighted images acquired at least 10 minutes after gadolinium injection [41]. The procedure for viability assessment using DE-MRI is relatively simple and can be performed in a single brief examination without the use of contrast and does not require pharmacologic or physiologic stress [85]. Furthermore, DE-MRI has been shown to predict segmental functional recovery as well as improvement in global function after reperfused acute MI in several studies [86, 87]. Moreover, DE-MRI has the ability to predict response to myocardial revascularization in patients who have established coronary artery disease [88, 89].  Figure 1 shows the role of MRI in the prediction of viability after myocardial infarction [90].

DE-MRI has been found to be comparable to each of DSE, SPECT, and PET in several studies [91–93]. However, DE-MRI is superior to DSE for viability determination in patients with poor endocardial border definition and in patients with atrial fibrillation [92]. Moreover, combination of different CMR parameters (a nonviability test delayed gadolinium enhancement and a viability test (inotropic stimulation with dobutamine) seems to be the optimal combination to assess hibernating myocardium. However, absence of scar or in presence of scar with <50% transmurality, DE-MRI alone seems to be enough without exposing the patient to additional stress testing [94].

### 5.7. Computed Tomography (CT)

Although using contrast-enhanced CT to assess viability is not new, recent advances in its temporal and spatial resolution with multidetector CT (MDCT) technology have gained interest for this application [95]. Recent studies revealed MDCT late enhancement (MDCT-LE) protocol is a reliable technique to detect necrotic and scarred myocardial tissue [96]. In addition, its usefulness for identification and characterization of infarcted myocardium in patients with recent to chronic MI has been shown by Chiou et al. [97] compared to rest-redistribution thallium SPECT, and DSE.

### 5.8. Electromechanical Mapping

Electromechanical endocardial mapping using a nonfluoroscopic catheter-based system (NOGA) was first described in 1996 [98]. Because myocardial ischemia and infarction have significantly different endocardial electrograms, the amplitude of the unipolar electrogram has been proposed as an indicator of myocardial viability [99]. Infarct size measured by electromechanical mapping compares well with pathology, echocardiography and SPECT images, and the boundary between normal and infarcted myocardium can be identified precisely by both electrical and mechanical patterns [100]. Early clinical studies of patients with left ventricular dysfunction undergoing revascularization suggest that electromechanical mapping is able to predict recovery of regional function [101, 102].

## 6. Endpoints in Viability Studies

Prior studies that evaluated the role of noninvasive imaging techniques in the detection of myocardial viability have focused on several clinical endpoints. These endpoints include: improvement in regional LV function (segments), improvement in global LV function (LVEF), improvement in symptoms (New York Heart Association [NYHA] functional class), improvement in exercise capacity (metabolic equivalents), reverse LV remodeling (LV volumes), prevention of sudden death (ventricular arrhythmias), and long-term prognosis (survival). Improvement in function after revascularization is still considered the final proof of viability [103]. From the clinical point of view, improvement in global LV function may be more important than improvement in regional function. A recent pooled data focused on viability assessment demonstrated that 53% of dysfunctional segments showed improvement in function after revascularization. Of these segments, 84% were considered to be viable according to the imaging modalities [104]. The LV EF has been demonstrated to be a very powerful predictor of prognosis. However, the majority of imaging studies that focused on viability assessment have evaluated only segmental improvement rather than global function improvement [103].

The proportions of viable segments needed for improvement in the LV EF differed among the studies. The available evidence (mainly using DSE) suggests that 20%–30% of the left ventricle needs to be viable to result in improvement in the LV EF [104]. It is also important to consider how great the improvement in LVEF must be to be clinically meaningful. Most studies have considered an improvement of 5% as significant, but this is mainly because of the inter-study reproducibility of measurements of ejection fraction rather than because this value is known to be clinically significant [38]. Recently, The Carvedilol Hibernation Reversible Ischemia (CHRISTMAS) trial showed that patients with more hibernating myocardium (identified by echocardiography and Tc99 m sestamibi) had a greater increase in LVEF on carvedilol treatment [105, 106]. This is different from what was reported in other reports and it could be a reflection of the optimization of patient management [107].

Another important endpoint in viability assessment is the prediction of LV remodeling, by comparing LV volumes before and after revascularization. Large trials with ACE inhibitors have shown that reverse LV remodeling is associated with improved survival. On the other hand, patients with predominantly scar tissue exhibited adverse LV remodeling, shown as an increase in both LV end-systolic and end-diastolic volumes. Therefore, surgery for patients with predominantly scar tissue did not result in reverse LV remodeling during followup [103]. Udelson et al. [108] conducted a substudy of the Occluded Artery Trial (OAT), which enrolled 124 OAT patients who underwent resting nitroglycerin-enhanced technetium-^99 m^ sestamibi SPECT before OAT randomization, with repeat imaging at 1 year. There were no significant differences in 1-year end-diastolic or end-systolic volume change between those with severely reduced versus moderately retained viability, or when compared by treatment assignment (angioplasty versus medical). In multivariable models, increasing baseline viability independently predicted improvement in LV EF (*P* = .005) but there was no interaction between viability and treatment assignment for any measure of LV remodeling [108].

## 7. Comparison of Imaging Techniques for Detection of Myocardial Viability

In the viability cascade, the areas with preserved response to dobutamine indicate a mild level of damage, which will usually allow adequate restoration of function following revascularization. For presumably more severe levels of damage, myocardial segments may be unresponsive to inotropic stress by dobutamine, but still can take up a significant amount of thallium. This is likely corresponding to a more advanced form of cellular damage, so that only those cellular functions that are strictly essential to cell survival (membrane integrity) are preserved [109].

**Figure 2:**
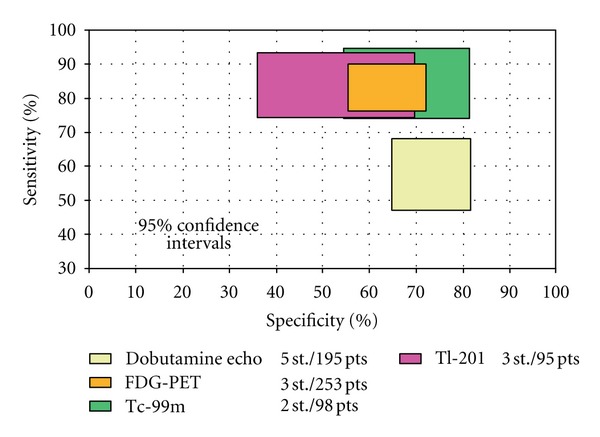
Comparison of sensitivities and specificities with 95% confidence intervals of the various techniques for the prediction of recovery of global LV function after revascularization (with permission from Schnikel et al. [103]).

**Figure 3:**
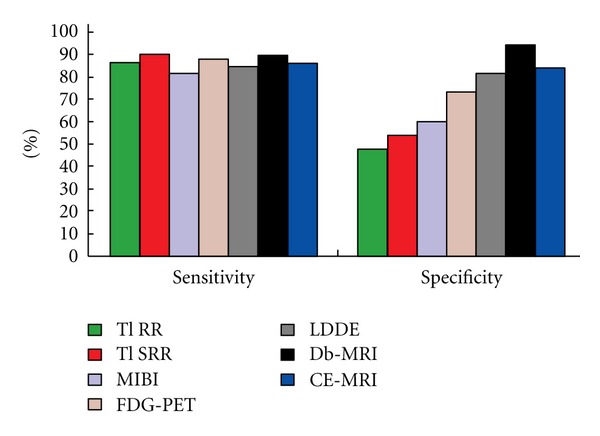
Sensitivity and specificity of thallium rest redistribution (Tl RR), Tc-99m sestamibi (MIBI), FDG-PET, low-dose dobutamine echocardiography (LDDE), dobutamine MRI (Db-MRI) and contrast-enhanced MRI (CE-MRI) for the prediction of viability (with permission from Marwick T (2003) Heart, BMJ Publishing Group, British cardiac society [110]).

In a recent meta-analysis, all available studies of regional left ventricular function in patients with ischemic left ventricular dysfunction before and after revascularization were pooled [103]. This analysis confirmed and extended the findings of the previous pooled analysis by the same group [104]. FDG-PET had the highest sensitivity, followed by nuclear SPECT imaging. In general, the nuclear imaging techniques had a higher sensitivity and lower specificity than DSE. Regarding prediction of global function improvement, DSE appeared to have the higher specificity, but the differences between techniques were not statistically significant (Figure 2) [103]. Marwick [110] analyzed the sensitivity and specificity of different imaging modalities in addition to MRI, modified from several meta-analyses and from direct comparison in individual patients. The analyses suggest that the accuracy of the common non-invasive tests is similar, with DSE being a little less sensitive but rather more specific than the competing modalities. Stress imaging with MRI has shown similar accuracy to DSE for identification of ischemia as well as assessment of viable myocardium, particularly in the setting of technically difficult echocardiography studies (Figure 3) [110]. The generally accepted opinion that SPECT and PET demonstrate higher sensitivity is confirmed in another meta-analysis [38]. Sensitivity and specificity of thallium rest redistribution, Tc-99m sestamibi (MIBI), FDG-PET, low dose dobutamine echocardiography, dobutamine MRI and contrast enhanced MRI for the prediction of viability are shown in Figures 2 and 3.

## 8. Viability and Prognosis

**Figure 4:**
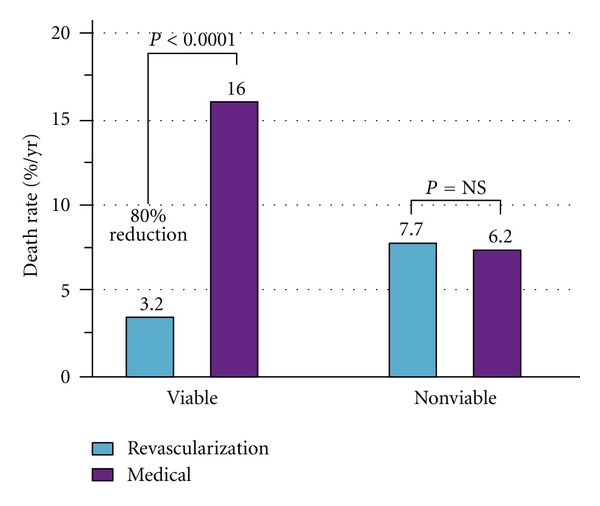
Analysis of pooled data from 24 studies using different viability techniques (with permission from Allman K et al (2002) J Am Coll Cardiol, Elsevier, Inc., [111]).

Generally, the final endpoint in viability studies is the long-term prognosis. Several studies have evaluated the prognostic value of viability in relation to therapy. These studies consistently showed better prognosis in patients who had viable myocardium and were revascularized, suggesting that revascularization stabilizes the unstable substrate of dysfunctional but viable myocardium [103]. Allman et al. [111] performed a meta-analysis of 24 prognostic studies that used various viability techniques and showed a 3.2% annual death rate in patients who were considered to have viable myocardium and who underwent revascularization, compared with a 16% annual death rate in patients who had viable myocardium but were treated medically (Figure 4). Similar findings were reported as well in a meta-analysis [103].

It should be noted, however, that medical therapy was not standardized in the studies analyzed by Allman and colleagues [111] and the adherence to optimal therapy was not adequately described. In the last decade, the medical treatment of heart failure has continued to improve and significant advances have been made in the techniques for coronary revascularization which have reduced intra-procedural and periprocedural risks [112].

Consequently, Camici and coworkers [107] pooled the data from 14 nonrandomized studies. They found a trend for a survival benefit in patients with CAD and LV dysfunction, with viable myocardium, who underwent revascularization compared with patients with viable myocardium treated medically. In the absence of viable myocardium, no clear-cut difference can be observed between treatments despite the fact that advances in both modalities of coronary revascularization procedures have reduced intra-procedural and peri-procedural risks. Most of these studies were based on retrospective analysis. On the contrary, reviewing the most recent literature, it was observed that the annual mortality rate in patients treated medically appears to be similar regardless of the presence of viability [113].

## 9. Outcome Studies

In a prospective study of 167 patients studied with FDG-PET, Desideri et al. [114] reported that the risk of cardiac death is increased only when the extent of viable tissue exceeds 20% of the LV, and together with the presence of left bundle branch block, it is an independent predictor of mortality. Observational studies in small cohorts of patients have highlighted that a long waiting time between assessment of viability and revascularization affected both the postoperative recovery of function and survival [115]. The impact of the time of revascularization on prognosis has recently been highlighted by Tarakji et al. [116] who assessed viability with PET scan in the largest prospective cohort of 765 consecutive patients. The investigators concluded that early intervention might be associated with reduced mortality from all causes.

The Heart failure Revascularization trial (HEART) is a multicenter study of 800 patients with heart failure, LV EF <35% and evidence of CAD who are receiving optimal medical treatment followed for 5 years. The main aim was to determine whether surgical revascularization improves the survival of patients who have evidence of dysfunctional but viable myocardium [117]. Only 138 of the planned 800 patients were enrolled because of withdrawal of funding due to slow recruitment. The investigators concluded that conservative management strategy may not be inferior to revascularization in patients with heart failure, LV systolic dysfunction, and extensive myocardial viability. However, this study was underpowered and recommended that further, larger trials to be done.

In a substudy from STICH trial [113] 601 patients with CAD and LV dysfunction were enrolled in a randomized trial of medical therapy with or without CABG, using SPECT, DSE, or both to assess myocardial viability. Of these patients, 298 were randomly assigned to receive medical therapy plus CABG and 303 to receive medical therapy alone. About one third of patients with viable myocardium and half of those without viable myocardium died (*P* = 0.003). However, after adjustment for other baseline variables, this association with mortality was not significant (*P* = 0.21). There was no significant interaction between viability status and treatment assignment with respect to mortality (*P* = 0.53). The study concluded that although the presence of viable myocardium was associated with a greater probability of survival in patients with CAD and LV dysfunction, however the assessment of myocardial viability did not recognize patients who can benefit from CABG, as compared with medical therapy alone. This finding may reflect the low rates of death among patients with viable myocardium who received medical therapy alone in STICH study (~7% per year), as compared with previously reported rates [113].

Recently, Gerber et al. studied 144 patients with coronary artery disease and myocardial dysfunction and concluded that detection of functional viable myocardium by DE-CMR is an independent predictor of mortality in patients with ischemic LV dysfunction before revascularization. This observation may be useful for preoperative selection of patients for revascularization [118].

## 10. Conclusion

In many of patients with CAD, the extent of remaining viable tissue is of clinical and prognostic significance. It can help to decide between revascularization and cardiac transplantation. Many subjects with heart failure and underlying coronary artery disease have an important amount of viable but dysfunctional myocardium, where myocardium keeps the ability to contract if perfusion improves. The dysfunctional viable myocardium has unique characteristics which form the basis for the different imaging modalities that are currently available for the assessment of myocardial viability. These modalities include different scintigraphic techniques, DSE, and recently MRI and CT modalities. Recent studies showed that the presence of viable myocardium was associated with a greater likelihood of survival in patients with CAD and LV dysfunction, but the assessment of myocardial viability did not identify patients with survival benefit from CABG, as compared with medical therapy alone. Assessment of myocardial viability alone should not be the deciding factor in selecting the best therapy. Whether the method of viability assessment or the underlying myocardial pathology and response, the determinant of optimal and appropriate mode of treatment is still debatable and needs more evidence.
